# OSU-03012 sensitizes breast cancers to lapatinib-induced cell killing: a role for Nck1 but not Nck2

**DOI:** 10.1186/1471-2407-13-256

**Published:** 2013-05-24

**Authors:** N Winston West, Aileen Garcia-Vargas, Charles E Chalfant, Margaret A Park

**Affiliations:** 1 University of Richmond, 28 Westhampton Way, Richmond VA, 23173, USA; 2 Virginia Commonwealth University, Department of Biochemistry, Cell and Molecular Biology, Sanger Hall, 1101 E. Marshall St., Richmond VA, 23298, USA; 3 Virginia Commonwealth University, Massey Cancer Center, 401 College Street, Richmond, VA 23298, USA; 4 Hunter Holmes McGuire VAMC, 1201 Broad Rock Blvd., Richmond VA, 23249, USA

**Keywords:** Breast cancer, Lapatinib, Combination therapy, Nck, eIF2-alpha

## Abstract

**Background:**

Lapatinib is characterized as an ErbB1/ErbB2 dual inhibitor and has recently been approved for the treatment of metastatic breast cancer. In this study, we examined mechanisms associated with enhancing the activity of lapatinib via combination with other therapies.

**Methods:**

In the present studies, estrogen receptor (ER) positive and ER negative breast cancer cells were genetically manipulated to up- or downregulate eIF2-alpha, its phospho-mutant, Nck1, or Nck2, then treated with OSU-03012, lapatinib or the combination and assayed for cytotoxicity/cytostaticity using clonogenic assays.

**Results:**

Treatment of breast cancer cell lines with lapatinib and OSU-03012 (a small molecule derivative of the Cox-2 inhibitor celecoxib) induced synergistic cytotoxic/cytostatic effects. This combination therapy corresponded to an increase in the phosphorylation of eIF2-α at serine^51^ and a decrease in Nck1 expression. Ectopic expression of phospho-mutant eIF2-α (Ser^51^Ala) or downregulation of eIF2-α in addition to downregulation of the eIF2-α kinase PERK inhibited the synergistic and cytotoxic effects. Furthermore, ectopic expression of Nck1, but not Nck2 abolished the decrease in cell viability observed in combination-treated cells. Downregulation of Nck1 failed to “rescue” the ablation of the cytotoxic/cytostatic effects by the phospho-mutant of eIF2-α (Ser^51^Ala) demonstrating that Nck1 downregulation is upstream of eIF2-α phosphorylation in the anti-survival pathway activated by lapatinib and OSU-03012 treatment. Finally, co-immunoprecipitation assays indicated that eIF2-α dissociates from the Nck1/PP1 complex after OSU-03012 and lapatinib co-treatment.

**Conclusions:**

These data indicate that OSU-03012 and lapatinib co-treatment is an effective combination therapy, which functions to enhance cell killing through the Nck1/eIF2 complex. Hence, this complex is a novel target for the treatment of metastatic breast cancer.

## Background

Breast cancer is currently the second most common cause of death due to cancer among women and leads to approximately 8,000 to 10,000 deaths per year [1]. Metastasis is the main cause of breast cancer related deaths, and these metastases are only poorly controlled with first generation therapies such as taxanes [2-4]. Both the ErbB2 and the ErbB1 receptors, members of the epidermal growth factor receptor (EGFR) family, are upregulated in many types of cancer, and overexpression of these proteins is associated with a greater likelihood of metastasis. Hence, this receptor family is a current therapeutic target for the treatment of metastatic breast cancer.

The epidermal growth factor receptor family comprises four members known as EGFR (ErbB1), Her2 (ErbB2), ErbB3, and ErbB4. Homo- and hetero-dimerization of these tyrosine kinase receptors occurs as a result of binding by various growth factors such as epidermal growth factor (EGF), after which cytoplasmic tail tyrosine residues are phosphorylated [5,6]. Phosphorylation leads downstream to the activation of various signaling cascades such as the extracellular-regulated kinase (ERK), and the Akt kinase cascades. These cascades lead to propagation of both survival and death signals [7,8]. Recently, lapatinib (Tykerb, GSK), an ErbB1/2 inhibitor, was approved for the treatment of metastatic breast cancer, as lapatinib is implicated in better outcomes in patients with metastases. Unfortunately, outcomes are still not ideal for patients with metastatic disease [9,10]. Thus therapies which enhance lapatinib-induced cell killing are needed in the clinic.

One possibility for combination therapy with lapatinib is the small molecule inhibitor, OSU-03012. This novel Celecoxib derivative induces death in cancer cells from multiple lineages without inhibiting Cox-2 [11-14]. Previous analyses indicate that OSU-03012 induces cell death partially via the activation of ER stress proteins including PKR-like ER kinase (PERK). PERK is a direct kinase of the eukaryotic initation factor 2 (eIF2) and phosphorylates this protein at the serine^51^ residue of the alpha subunit [15,16]. Phosphorylation of eIF2-α leads to increased expression of the pro-apoptotic transcription factor CHOP as well as the expression of HSP70 family chaperones. Our previous analyses demonstrated that OSU-03012 reduced Grp78/BiP levels and increased HSP70 levels in a PERK-dependent fashion [11,12]. The laboratory of Dr. Chen, in general agreement with our previous studies, has shown that inhibition of ErbB1 in ErbB1-addicted NSCLC enhances the toxic effects of OSU-03012, and that this is in part due to increased ER stress signaling and increased levels of DR5 [14]. The laboratory of Dr. Paul Dent has also recently published that OSU-03012 and lapatinib synergize in glioblastoma cell lines, although by a different mechanism than the one found in this manuscript [17].

In the current studies, we assessed whether OSU-03012-induced killing of breast cancer cell lines was enhanced by the addition of lapatinib. We show that a decrease in adaptor protein Nck1, but not Nck2, [18,19] is necessary for cell killing in both ER positive and ER negative breast cancer cell lines. Furthermore, we show that increased eIF2-α phosphorylation on Serine^51^ induced by the combination of OSU-03012 and lapatinib is responsible for the synergistic effects of these agents. Thus, the Nck1/eIF2 complex is identified in this study as a novel target for the treatment of metastatic breast cancer.

## Methods

### Cell culture

The MDA-MB-231 cell line (purchased from American Type Culture Collection, ATCC) and the BT474 cell line (ATCC) were maintained in RPMI (Invitrogen). ATCC published standards are recognized by the American National Standards Institute (ANSI) and are compatible with the requirements of the International Organization for Standardization (ISO). Both cell lines were supplemented with 10% fetal bovine serum (FBS, Invitrogen) and 1% Penicillin / Streptomycin (Invitrogen). All cell lines were maintained in a 95% air / 5% CO2 incubator at 37°C. Cells were passaged once every 3-5 days (~90% confluence), and all experiments were performed during the first 12 passages.

### Plasmids and reagents

eIF2-α expression plasmids were constructed by Ron et. al. and purchased from Addgene (plasmid numbers #21808 and 21807, [20]). GFP-tagged Nck1 and Nck2 plasmids were a generous gift from Dr. L. Larose [18,19]. Antibodies to Nck1, phospho-eIF2-α (Serine^51^), total eIF2-α, ERK, phospho-ERK, PTEN, phospho-PTEN, PP1, phospho-PP1 and β-actin were purchased from Cell Signaling Technologies. Nck2 antibodies were purchased from Novus Biologicals. siRNA molecules against Nck1 and mutant siRNA molecules were custom manufactured by Dharmacon. The sequence used was previously published by Dr. W. Li and colleagues [21]. A mutant sequence containing 9 mutations was also manufactured as a control to ensure specificity of knockdown. Sequences are as follows (single stranded, sense): siNck1 5’ GGC CTT CAC TCA CTG GAA A 3’; Mutant Nck1 5’ CGC TTC CAC TGC TGA GAG A 3’. Pre-designed and validated siRNA molecules to downregulate eIF2-α and control scrambled siRNA molecules were purchased from Qiagen. siRNAs targeting ATF6 and IRE-1 were generous gifts from the laboratory of Dr. Paul Dent.

### Apoptosis assays

Cells were treated as indicated. 24 - 48 hrs later, cells were trypsinized, washed and stained with Annexin V-PE and propidium iodide using the ApoScreen Annexin V Apoptosis Kit (Southern Biotech) according to manufacturer’s instructions. Cells were detected using a BD FACSCanto II and analyzed using the accompanying FACSDIVA software.

### Transfection (plasmid)

Plasmid transfections were accomplished using the Effectene system (Qiagen) according to manufacturer’s instructions. Briefly, plasmid DNA (1 μg) was incubated in the presence of EC buffer and a 150:18 dilution of the Enhancer reagent for 10 minutes followed by the addition of the Effectene reagent (at a 168:20 dilution). Plasmid samples were incubated for a further 10 minutes then diluted to 1 mL with complete medium and added by single drops to the sample. Cells were allowed to accumulate the recombinant proteins for 24-48 hours. All steps excluding the incubation of DNA, EC buffer, Enhancer reagent and Effectene reagent were undertaken in 10% FBS-containing medium.

### Transfection (siRNA)

siRNA transfections were performed using the Dharmafect 1 reagent (Dharmacon) according to manufacturer’s instructions. Briefly, siRNA molecules (25 nM final concentration) were incubated in serum- and antibiotic-free medium. Concurrently, 5 μL Dharmafect 1 reagent was incubated in serum- and antibiotic-free medium. Both tubes were incubated at room temperature for 10 minutes then combined and incubated at room temperature for an additional 20 minutes. siRNA was then added to cells one drop at a time. Cells were incubated for at least 48 hours to achieve downregulation of the target mRNA.

### Survival assays

Clonogenic assays were performed as previously described [22]. Briefly, cells were transfected and treated as indicated in the figure legends. Cells were then plated onto 6-well plates at a density of 200-400 cells / well and allowed to form colonies over the next 10-14 days. Colonies were stained using crystal violet stain, and cells that underwent ≥ 50 doublings were counted as a colony.

### Western blotting

Cells were plated, cultured and treated as indicated. Cells were washed 2 times in PBS and lysed using CelLytic (Sigma) lysis buffer supplemented 1:100 with protease and phosphatase inhibitors (Cell Signaling) and by sonication. Protein concentration was assessed using Bio-Rad protein assay reagent. Equal amounts (10-20 μg) of protein were subsequently electrophoresed on 10-12% SDS polyacrylamide gels and electrophoretically transferred to PVDF membranes (Bio-Rad). Membranes were blocked in PBS supplemented with 0.1% TWEEN-20 and 5% dry milk and exposed to primary and secondary antibodies as indicated. Membranes were developed using SuperSignal West reagents (Pierce).

### Co-immunoprecipitation assays

Cells were treated as described in figure legends. Cells were then harvested using NP-40 buffer (20 mM Tris-HCl, pH 8.0; 137 mM NaCl; 2 mM EDTA; 2% NP-40; protease and phosphatase inhibitor cocktail (added prior to use)). Lysate was pre-incubated with protein A/G agarose beads (1 h, 4°C with rotation). Concurrently, Protein A/G agarose beads (Pierce) were incubated with antibodies raised against either total eIF2-α or total PP1 (Cell Signaling). Beads were washed 3 times with NP-40 buffer and then added to cell lysates. Lysates + beads were incubated at 4°C for 4 – 16 h with rotation and washed 3 times in NP-40 buffer. Bound proteins were released from the antibody-coated beads using 200 mM glycine, pH 3.0. Electrophoresis and western blotting procedures were then performed as previously described.

### Isobologram analyses

Isobologram analyses were performed using the method of Chou and Talalay [23]. Briefly, colony formation assays were performed using stepwise increasing concentrations of OSU-03012 and lapatinib either singly or in combination (1 μM, 2 μM and 3 μM both in single and in combination treatments). Analyses were then performed using the Calcusyn program (Biosoft). Fraction affected (FA) was calculated and the combination index (CI) was then used as a measure of synergy.

### Statistics

All *P* values refer to paired student’s t-tests; differences with *p*≤0.05 were considered significant. Analyses were performed using the Sigmaplot software.

## Results and discussion

**Figure 1:**
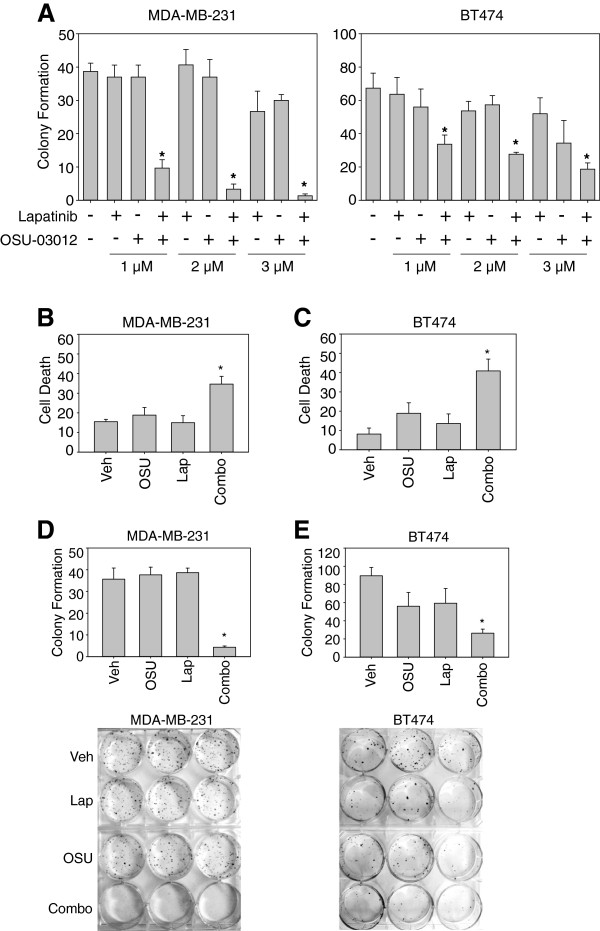
**OSU-03012 and lapatinib act to kill ER-positive and ER-negative breast cancer cells in combination. A, D-E**: ER-positive BT474 and ER negative MDA-MB-231 cell lines were treated with the indicated concentrations of OSU-03012 and lapatinib (**A**) or 2 μM lapatinib OSU-03012 and 2 μM lapatinib (**D-E**) for 48 h. Cells were then singly plated onto 6-well plates to assay for clonogenic capacity. **B-C**: BT474 and MDA-MB-231 cells were treated with 2 μM OSU-03012 (OSU) or 2 μM lapatinib (Lap) or the combination, incubated for 48 h, then assayed using Annexin V/PI for cell death. All measurements are ± SEM. * indicates a p < 0.05 when compared to the vehicle-treated condition.

*OSU-03012 and lapatinib synergize to induce cell death in both ER positive and ER negative breast cancer cell lines*. As stated previously, one possibility for combination therapy with the FDA-approved drug lapatinib is the small molecule OSU-03012 as this novel Celecoxib derivative induces cell death in cancer cells from multiple lineages [11-14]. In our initial studies, cell death (via annexin V-PE) of MDA-MB-231 (ER negative, [24]) and BT474 (ER positive, [24]) breast cancer cells was assessed after co-treatment with OSU-03012 and lapatinib. Neither OSU-03012 nor lapatinib at 1 or 2 μM (well below the maximum tolerated dose) induced significant increases in cell death when compared to control conditions (Figure 1A-C). However, treatment of BT474 cells with single agents at 3 μM resulted in decreases in clonogenic capacity when compared to controls (Figure 1A). Treatment with the combination at all concentrations tested showed a greater than additive effect (See Table 1, Figure 1). This effect was confirmed by repeating the experiment and demonstrating a decrease in the survival of cells treated with the combination at 2 μM (see Figure 1D-E). Synergy was confirmed by survival assays followed by isobologram analyses (Table 1, [23]). A combination index (CI) value of less than 1 indicates synergistic effects, whereas a CI value of 1 indicates an additive effect and a CI value of greater than 1 indicates antagonistic effects. These data demonstrate that OSU-03012 and lapatinib act synergistically to induce cell death in both ER positive and ER negative breast cancer cell lines and provided a rationale for treatment of cell lines at 2 μM for the remainder of the studies.

**Table 1:** Isobologram analysis of MDA-MB-231 and BT474 cell lines indicates that the drugs are synergistic in multiple breast cancer lines

**Drug Conc lap**	**Drug Conc. OSU**	**FA**	**CI value**
1 uM	1 uM	.40	.12
2 uM	2 uM	.41	.24
3 uM	3 uM	.31	.24
BT474			
1 uM	1 uM	.5	.35
2 uM	2 uM	.41	.52
3 uM	3 uM	.28	.48

Interestingly, OSU-03012 and lapatinib combination therapy was more effective against MDA-MB-231 cells than BT474 cells. Therefore, our findings argue that targeting ER stress proteins may increase the efficacy of traditional therapies specifically for metastatic breast cancers [11-13] since the BT474 cell line is less invasive than the triple negative MDA-MB-231 cell line [25,26]. Specifically, we found a greater decrease in cell viability and a lower CI value for synergy between OSU-03012 and lapatinib in the triple negative cell line MDA-MB-231 (harvested from the metastatic pleural ascites) than in ErbB2-amplified BT474 cell line (harvested from a primary site). These findings provide support for the hypothesis that OSU-03012 and lapatinib in combination may be more effective against metastatic breast cancers than non-metastatic breast cancers. These results are also in line with recent studies by Sanz-Pamplona et. al., which showed that upregulation of GRP94, an ER stress protein, is an effective marker for brain metastases of breast cancers [27], and others [28], which showed that other ER stress markers are upregulated during suspension conditions.

Our data demonstrating that MDA-MB-231 cells are more sensitive to the combination of OSU-03012/lapatinib are also in general agreement with the findings in Figure 7B, that PP1 associates significantly less with eIF2-α after OSU/lapatinib treatment in MDA-MB-231 cells than in BT474 cells. While PTEN, Raf, and Akt levels and mutation status appear to be similar in both MDA-MB-231 and BT474 cells [29-31], BT474 cells express a constitutively active form of PI3KCA (K111N), in addition to overexpressing ErbB2 [32]. It may be that upregulation of the PI3K/Akt pathway represents a potential pathway of resistance for cell lines treated with OSU-03012/lapatinib in combination. Therefore, inhibitors of the PI3K pathway should be combined with OSU-03012/lapatinib in future studies.

**Figure 2:**
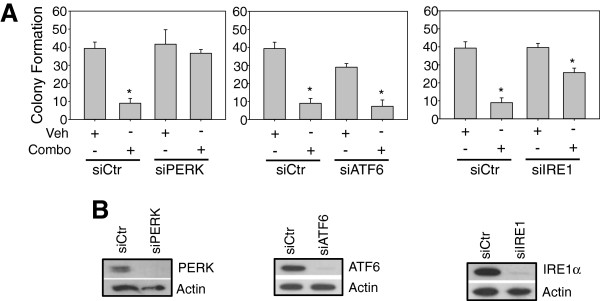
**ER stress via PERK activation may be responsible for lapatinib/OSU-03012-induced cytotoxicity/cytostaticity. A-B**: MDA-MB-231 cells, 24 h after plating, were transfected with the indicated siRNA. After a 24 h incubation, cells were either plated singly onto 6-well plates and allowed to attach overnight (**A**) or harvested for immunoblotting to ensure knockdown (**B**). Cells in (**A**) were treated with vehicle or OSU-03012/lapatinib (48 h) then media was replaced and colonies were allowed to develop over the next 10-14 d. Colonies were counted using crystal violet stain and the number of colonies was graphed (n=3, *=p<0.05).

**Figure 3:**
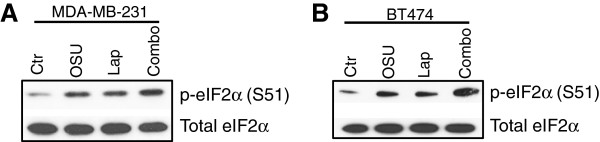
**Phosphorylation of eIF2-α indicates ER stress signaling in MDA-MB-231 and BT474 cells after treatment with OSU-03012 and lapatinib. **MDA-MB-231 cells and BT474 cells (1 x 10^6^) were subjected to vehicle (DMSO, Ctr), OSU-03012 (2 μM), lapatinib (2 μM) or the combination as indicated for 3 h. Cells were then lysed and protein extracts (10-20 μg) were subjected to SDS PAGE followed by western immunoblotting for the indicated proteins.

*Phosphorylation of eIF2-α at serine*^*51*^*specifically induces cell death in response to OSU-03012 and lapatinib via protein phosphatase-1.* Previous analyses indicate that OSU-03012 induces cell death partially via the activation of ER stress proteins, including PKR-like ER kinase (PERK, [14] see Figure 2), and that the ER stress response is important in breast cancer tumorigenesis [27,28]. We therefore determined whether downregulation of the three main ER stress sensors (PERK, IRE-1α and ATF6) decreased cell death induced by OSU-03012 and lapatinib in combination. The involvement of PERK in lapatinib/OSU-03012-induced cytotoxicity was confirmed in these studies. Other ER stress sensors did not protect against lapatinib/OSU-03012-induced cytotoxicity/cytostaticity (ATF6), or had a small protective effect (IRE-1α, see Figure 2). We therefore chose to focus on PERK-mediated effects for the remainder of these studies. PERK is a direct kinase of the eukaryotic initation factor 2 (eIF2), phosphorylating this protein at the serine^51^ residue of the alpha subunit [15]. Thus, the phosphorylation state of eIF2-α was assessed in these studies as an indicator of ER stress. Surprisingly, treatment of breast cancer cells with OSU-03012 or lapatinib alone only affected the phospho-state of eIF2-α on Ser^51^ in a minor fashion (Figure 3). Importantly, the phosphorylation of this protein was increased significantly after co-treatment lapatinib and OSU-03012.

**Figure 4:**
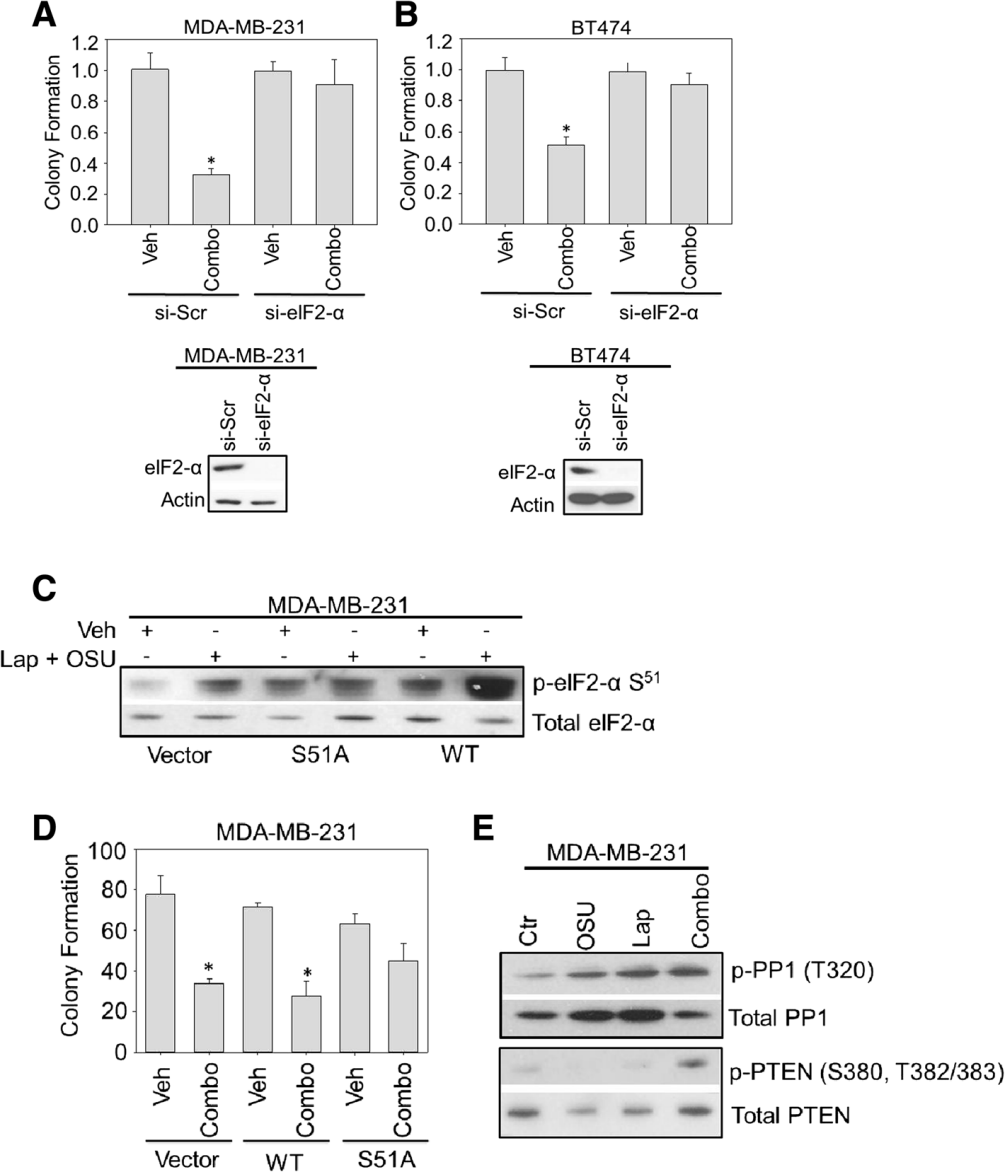
**The role of eIF2-α phosphorylation in cell death induced by OSU-03012 and lapatinib. A-B**: MDA-MB-231 (**A**) or BT474 (**B**) cells (5 x 10^5^) were transfected with the indicated siRNA molecules and incubated for 48 h. Cells were then treated with either vehicle (Veh) or the combination of OSU-03012 (2 μM) and lapatinib (2 μM) (combo) as indicated and either subjected immunoassays (bottom panels) or plated for clonogenic capacity (top graphs). Numbers indicated are percent control (e.g. Veh). **C-D**: MDA-MB-231 cells (5 x 10^5^) were transfected with a control vector (Vector), wild-type (WT) or the Ser^51^Ala mutant (S51A) eIF2-α plasmids. After a further 24 h cells were plated onto 6-well plates to assay for clonogenic capacity (**D**) or subjected to immunoblotting as described in Materials and Methods (**C**). Cells were treated with either vehicle (Veh, DMSO) or OSU-03012 (2 μM) and lapatinib (2 μM) in combination (combo) for 24 h (**D**) or 3 h (**C**). Colonies were allowed to develop for the next 10-14 days. * indicates a p < 0.05 with respect to vehicle-treated cells. **E**: MDA-MB-231 cells (1 x 10^6^) were plated and treated with the indicated drugs (Vehicle (Ctr, DMSO), OSU-03012 (OSU, 2 μM), lapatinib (Lap, 2 μM) or the combination (Combo)). Three hours later, cells were harvested and subjected to immunoblotting. Samples were probed with the indicated antibodies (see Materials and Methods).

Since eIF2-α phosphorylation on Ser^51^ was upregulated by combination therapy (Figure 3), the role of eIF2-α was examined in the synergistic killing of breast cancer cells. As shown in Figure 4A and B, knockdown of eIF2-α completely ablated the decrease in survival induced by OSU-03012 and lapatinib. Importantly, ectopic expression of the inactive Ser^51^Ala phospho-mutant attenuated cell death induced by the combination treatment in contrast to ectopic expression of wild-type eIF2-α (Figure 4C and D). These data demonstrate that eIF2-α phosphorylation on serine^51^ is a central event in the induction of cell death induced by OSU-03012 and lapatinib.

PTEN [33] and protein phosphatase 1 (PP1, [34]) are two phosphatases whose activities are linked to eIF2-α phosphorylation. Thus, we assessed the activity of these phosphatases as upstream determinants of OSU-03012/lapatinib-induced eIF2-α phosphorylation. First, the phospho-status of PTEN was examined as an indicator of activation, but no increases were observed for the phosphorylation of PTEN (Figure 4E). Instead, the phosphorylation pattern was similar to the pattern of total PTEN expression. Hence, enhanced PTEN activity is unlikely affecting OSU-03012- and lapatinib-induced cell death/reduced survival. In Figure 4E, we observed that the phosphorylation of the PP1 was significantly increased indicating a decrease in the activity of PP1 (Figure 4E, [34]). Thus, with regards to upstream events leading to eIF2-α activation, PP1, but not PTEN, is a likely candidate responsible for the dephosphorylation of eIF2-α induced by OSU-03012/lapatinib in combination.

Taken together, the data in Figures 3 and 4 showed that OSU-03012/lapatinib in combination upregulated ER stress-related pathways, and that downregulation of eIF2-α phosphorylation at serine^51^ completely ablated cell death induced by OSU-03012/lapatinib and demonstrated that PP1 was a likely candidate for eIF2-α dephosphorylation.

ER stress aggravators (ERSAs) are a relatively recent addition to our arsenal of therapeutic agents for the treatment of cancer. There are multiple reports [27,28,35] that ER stress factors are upregulated in many types of cancer suggesting that these pathways may be ones to which cancers may become addicted and therefore represent good targets for treatment. OSU-03012 represents one ERSA which may be used to enhance ER stress pathways in cancer cells. This may activate a response in which the cancer cell shifts from using ER stress signaling as a survival mechanism to an apoptotic one. Our findings demonstrate that eIF2-α phosphorylation is a major event in the cell death pathways induced during treatment with OSU-03012/lapatinib. Furthermore, the question whether other molecules that induce ER stress will also enhance lapatinib-induced cell killing should be pursued in light of these studies.

### Nck1, but not Nck2 is intrinsic to OSU-03012/lapatinib-induced cell death

**Figure 5:**
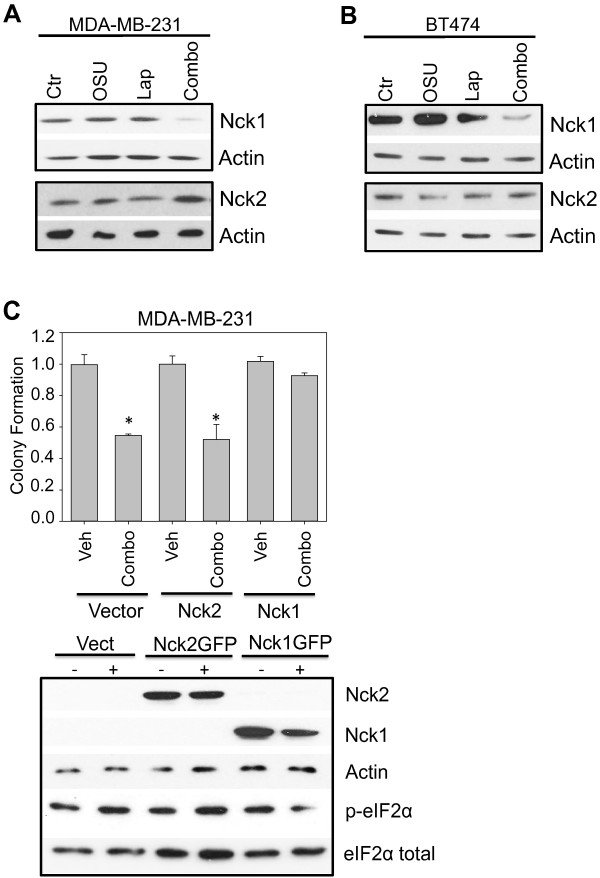
**Upstream signaling responsible for eIF2-α phosphorylation: A role for Nck1. A-B**: MDA-MB-231 cells and BT474 cells were treated with vehicle (Ctr, DMSO), OSU-03012 (OSU, 2 μM), lapatinib (Lap, 2 μM) or the combination (Combo) for 3 h, then harvested for immunoblotting assays as described in Materials and Methods. Membranes were probed with the indicated antibodies. **C**: MDA-MB-231 cells (5 x 10^5^) were transfected with plasmids to express either GFP-Nck1 or GFP-Nck2 as described. After an additional 24 h, cells were treated with the combination of OSU-03012 and lapatinib as indicated for 24 h (upper graphs) or 3 h (lower panels), and then either plated onto 6-well dishes and allowed to form colonies (graphs represent percent control) or harvested for immunoblotting assays and probed with the indicated antibodies. * indicates a p<0.05.

PP1 has been found by Larose et al [18,19] in a complex containing both eIF2 and the protein Nck1. Nck1 (or Nckα), an SH-only adaptor protein, was originally characterized as playing a role in driving cell motility [36], a hallmark of metastatic cancer. Nck1 binds to eIF2-β, preventing the phosphorylation of eIF2-α specifically on Serine^51^, and dissociation of Nck1 leads to increased levels of eIF2-α phosphorylation. Thus, we examined the role of Nck1 in the enhanced phosphorylation of eIF2-α on Serine^51^. A robust, greater-than-additive decrease in the levels of Nck1 was observed in combination-treated samples (See Figure 5A,B) in contrast to cells treated with a single drug. Nck2 (also known as Nckβ) expression did not follow the same pattern indicating a novel differential role for these two family members in OSU-03012- and lapatinib-induced cell killing.

**Figure 6:**
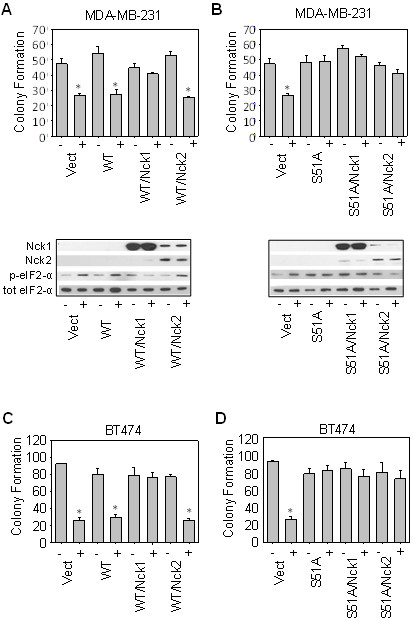
**Nck1, but not Nck2 expression ablates the increase in cell death induced by OSU-03012 and lapatinib. A-D**: MDA-MB-231 cells (**A, B**), or BT474 cells (**C, D**) (5 x 10^5^) were co-transfected with either wild-type eIF2-α and the two Nck isoforms (**A, C**), or the S^51^A phospho-mutant and the two Nck isoforms (**B, D**). Cells were allowed to incubate for 24 h to induce ectopic expression, and then treated with either vehicle (indicated with a -), or the combination of OSU-03012 and lapatinib (2 μM each, indicated with a +). Cells were either harvested after a 3 h treatment for western blotting as described in Materials and Methods (bottom panels), or plated singly onto 6-well plates to assay for clonogenic capacity (upper graphs). Cells were treated with OSU-03012 (2 μM) and lapatinib (2 μM) for 24 h, and then allowed to form colonies for 10-14 days. Total colony counts were graphed. * denotes a p-value of <0.05.

Next, we examined the role of Nck1 in the cell death and eIF2 Ser^51^ phosphorylation induced by the combination of OSU-03012 and lapatinib. The decrease in both clonogenic capacity and eIF2-α phosphorylation in MDA-MB-231 cells after OSU-03012 and lapatinib combination treatment was “rescued” by the ectopic expression of Nck1 (see Figure 5C), but not by ectopically expressing Nck2. Furthermore, Nck1, when co-expressed with wild-type eIF2-α, ablates the increase in cell death induced by OSU-03012 and lapatinib indicating a role in the same pathway for this protein (See Figure 6A and C). In contrast, ectopic co-expression of the Ser^51^Ala phospho-deficient mutant of eIF2-α with either Nck1 or Nck2 ablated all cell death induced OSU-03012 and lapatinib in combination (See Figure 6B and D). Co-expression of Nck2 and wild-type eIF2-α did not affect the levels of cell death indicating that this pathway is specific for Nck1.

**Figure 7:**
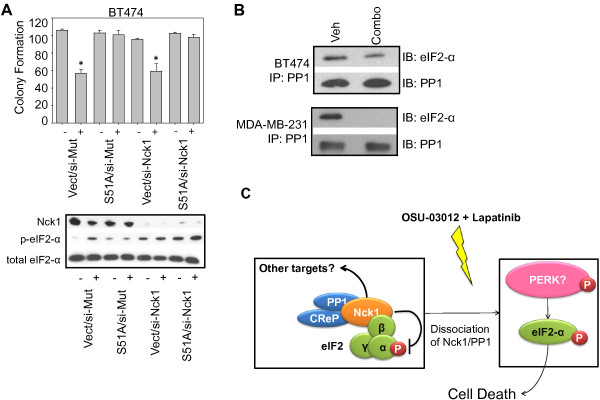
**Nck1 is upstream of eIF2-α phosphorylation in the cell death induced by the combination of OSU-03012 and lapatinib. A**: BT474 cells (5 x 10^5^) were co-transfected with the Ser^51^Ala eIF2-α mutant and GFP-Nck1. Cells were allowed to incubate for 24 h to induce ectopic expression then treated with either vehicle (indicated with a -), or the combination of OSU-03012 and lapatinib (2 μM each, indicated with a +). Cells were either harvested for western blotting as described previously (lower panel), or plated into 6-well plates to assay for clonogenic capacity as described previously (upper graph). Graphs represent total colony number. * denotes a p-value of <0.05. **B**: BT474 (upper panel) and MDA-MB-231 (lower panel) cells were treated with either vehicle (Veh) or OSU-03012 and lapatinib in combination (combo). PP1 was immunoprecipitated and the resulting membranes were immunoblotted with eIF2α and PP1. **C**: Our data indicate that Nck1 and PP1, which are originally in a complex with eIF2 are dissociated after treatment with the combination of OSU-03012 and lapatinib. This event frees eIF2-α to become phosphorylated by one of many upstream kinases such as PERK, leading to ER stress and eventual cell death.

Finally, in agreement with our hypothesis that decreased Nck1 expression is upstream to the increase in eIF2-α phosphorylation, we showed that downregulation of Nck1 was insufficient to re-sensitize BT474 cells to the ablation of OSU-03012 and lapatinib-induced cell death when the phospho-mutant of eIF2-α is ectopically expressed (Figure 7A). In addition, OSU-03012/lapatinib in combination induces a decrease in the association of eIF2-α with PP1 (Figure 7B). Taken together, these data demonstrate that a major mechanism of cell death via the combination of OSU-03012 and lapatinib is a decrease in Nck1 expression followed by upregulation of eIF2-α phosphorylation, and thus ER stress-related cell death (Figure 7C).

Larose and colleagues [18,19] found that Nck1 forms a complex with eIF2 and PP1. Dissociation of this complex can lead to eIF2-α phosphorylation at serine^51^ and a decrease in protein translation. eIF2-α may also be phosphorylated at serine^51^ by the ER resident kinase PERK during ER stress. Since we show in Figure 2 that OSU-03012/lapatinib in combination induces ER stress in part by PERK activation, we performed studies aimed at determining the role of Nck1 in ER stress-induced cell death by OSU-03012 and lapatinib in combination. Our studies showed that ectopic expression of Nck1 abolished the cell death induced by OSU-03012/lapatinib. Furthermore, upregulation of Nck1 “rescues” the cell death induced by wild-type eIF2-α overexpression. Thus, the studies reported here demonstrate that the Nck1/eIF2 complex is a key point at which lapatinib and OSU-03012 act to synergistically kill metastatic breast cancer cells, and generally support Larose’s findings that PP1 is important in this complex.

In contrast to our findings implicating a PP1, Nck1 and eIF2-containing complex in the cytotoxicity/cytostaticity induced by OSU-03012/lapatinib, the Dent laboratory has recently published that lapatinib enhances OSU-03012-induced cell killing in glioblastoma models and that this phenomenon occurs via an ErbB/Akt/PTEN pathway [17]. MDA-MB-231 and BT474 cells as well as GBM6 and GBM12 (used in [17]) cell lines are all PTEN wild-type. Therefore, cancer-type-specific pathways may be responsible for this apparent contradiction. Our data suggest that further experiments may need to take these cancer-specific differences into account when designing therapeutic regimens.

Recently, EGFR-mediated Nck1/Rap1 activation has been shown to upregulate metastasis in a model of metastatic pancreatic carcinoma without affecting primary tumor growth [37]. These findings raise two intriguing possibilities: 1) Nck1 downregulation may be a singularly efficacious inducer of cell death specifically for metastatic breast cancer cells, and 2) eIF2 may play a role in the metastatic process. We observe a small, but insignificant decrease in the viability of BT474 cells (a non-invasive cell line, see Figure 7) after RNAi-mediated inhibition of Nck1, which may be indicative that inhibition of Nck1 alone may induce cell death in more invasive cell lines. In addition, we observe that Nck1 is downregulated only with the combination treatment in MDA-MB-231 (a more invasive cell line) cells even though eIF2-α phosphorylation is upregulated in samples treated with single drugs. eIF-4E, the mRNA cap-binding protein essential for the initiation of translation, has been found to contribute to malignancy by enabling translation of select mRNAs that encode proteins involved in growth, angiogenesis, survival and malignancy [38]. Interestingly, ER stress signaling and eIF2-α phosphorylation have been linked to drug resistance and survival in occult dormant carcinoma cells [39]. However, eIF2-α has never before been characterized specifically as a regulator of metastasis. Therefore, studies aimed at characterizing the involvement of eIF2 in metastasis, both *in vivo* and *in vitro*, are a natural continuation of these findings as are studies aimed at examining the potential of Nck1 inhibition as a therapy specific for metastatic breast cancer.

## Conclusions

Combination therapies are especially useful in the treatment of many cancers, in part due to the ability of separate drugs to target multiple separate survival pathways upregulated in many cancer lineages [40]. In these studies, we have used the concept of combination therapies to delineate the interaction between OSU-03012 and lapatinib. We showed that OSU-03012 and lapatinib synergized to induce cell death in both an ER positive and an ER negative breast cancer cell line suggesting that this therapeutic model may be effective against a variety of breast cancer phenotypes. We also demonstrated that eIF2-α phosphorylation is a central event in the synergistic cytotoxicity/cytostaticity induced by the combination therapy of OSU-03012 and lapatinib, and that this event is partially mediated by the protein phosphatase PP1/Nck/eIF2 complex.

These studies describe a novel mechanism of cytotoxicity/cytostaticity via Nck1-mediated eIF2-α phosphorylation for the combination of lapatinib and OSU-03012. We conclude that OSU-03012 and lapatinib act synergistically to induce cell death via the downregulation of Nck1/PP1 and the subsequent dissociation of this complex from eIF2-α. We also conclude that this dissociation likely leads to a PP1-mediated enhancement of eIF2-α phosphorylation at serine^51^, a marker for ER stress and a central event in the induction of cell death by OSU-03012/lapatinib. This work additionally identifies the Nck1/PP1/eIF2-α as a novel target for inhibition for future therapies.

## Abbreviations

ErbB: Avian erythroblastosis oncogene B; ER: Estrogen receptor; eIF: Eukaryotic initiation factor; ERK: Extracellular-regulated kinase; PKR: Protein kinase R; PERK: PKR-like ER kinase; PP1: Protein phosphatase-1; DR5: Death receptor 5; PTEN: Phosphatase and tensin homolog; PBS: Phosphate buffered saline; FBS: Fetal bovine serum; PVDF: Polyvinylidene fluoride; FA: Fraction affected; CI: Combination index.

## Competing interests

The authors wish to declare that they have no competing interests.

## Authors’ contributions

NWW and AGV collected clonogenic and apoptosis data, and AGV performed siRNA experiments. MAP and CEC were involved in the experimental design and conception. MAP performed western blotting, siRNA and plasmid transfection, co-immunoprecipitation and some clonogenic assays. MAP analyzed the data and wrote the manuscript with editorial input from CEC. All authors read and approved the final manuscript.

## Pre-publication history

The pre-publication history for this paper can be accessed here:

http://www.biomedcentral.com/1471-2407/13/256/prepub
